# Arrays of Regenerated Fiber Bragg Gratings in Non-Hydrogen-Loaded Photosensitive Fibers for High-Temperature Sensor Networks

**DOI:** 10.3390/s91008377

**Published:** 2009-10-22

**Authors:** Eric Lindner, Christoph Chojetztki, Sven Brueckner, Martin Becker, Manfred Rothhardt, Johan Vlekken, Hartmut Bartelt

**Affiliations:** 1 Institute of Photonic Technology (IPHT), Albert-Einstein-Str. 9, 07745 Jena, Germany; E-Mails: sven.brueckner@ipht-jena.de (S.B.); martin.becker@ipht-jena.de (M.B.); manfred.rothhardt@ipht-jena.de (M.R.); hartmut.bartelt@ipht-jena.de (H.B.); 2 Fibre Bragg Grating Sensors (FBGS) Technologies GmbH, Buchaer Str. 6, 07745 Jena, Germany; E-Mail: cchojetzki@fbgs-technologies.com (C.C.); 3 Fibre Optic Sensors and Sensing Systems (FOS&S), Cipalstraat 14 - B-2440 Geel, Belgium; E-Mail: jvlekken@fos-s.com (J.V.)

**Keywords:** Bragg gratings, grating regeneration, temperature sensing

## Abstract

We report about the possibility of using regenerated fiber Bragg gratings generated in photosensitive fibers without applying hydrogen loading for high temperature sensor networks. We use a thermally induced regenerative process which leads to a secondary increase in grating reflectivity. This refractive index modification has shown to become more stable after the regeneration up to temperatures of 600 °C. With the use of an interferometric writing technique, it is possible also to generate arrays of regenerated fiber Bragg gratings for sensor networks.

## 1. Introduction

Fiber Bragg gratings are well established as strain and temperature sensors, not only in the field of structural health monitoring in civil engineering but also in the aerospace and medical industries. The advantages of fiber Bragg gratings are their compact size and the possibility of multiplexed sensors, even over long distances. For use as a temperature sensor, the induced refractive index modification is crucial. Recently, a new type of refractive index modification was introduced by Canning *et al.* [1,2], which leads to a regeneration of fiber Bragg gratings at temperatures of about 900 °C. This regenerative process was observed in germanium-boron-doped photosensitive fibers but only by applying hydrogen loading. We recently reported a similar process in germanium doped fibers but without any hydrogen loading and by using the 248 nm wavelength for grating inscription [3]. The regeneration process, carried out at lower temperatures and leads to temperature stabilities of up to 600 °C. By using an interferometric grating writing technique we can generate arrays of regenerated fiber Bragg gratings for high-temperature sensor networks. The concept of such array sensor structures is discussed in this paper.

## 2. Experiments

The writing setup for grating inscription is a modified Talbot interferometer, which was introduced first by Dockney *et al.* [4] and the performance of which was later demonstrated with different writing laser sources [5,6]. We used a KrF excimer laser at 248 nm wavelength together with a highly germanium doped photosensitive fiber for grating generation (18 mol. % GeO_2_). The energy density of the writing laser was adjusted by moving a cylindrical lens in front of the fiber and was calculated by using simple geometric optics equations [3]. The fluence as the product of energy density and number of pulses was varied for investigating the parameters of the effect.

**Figure 1.**
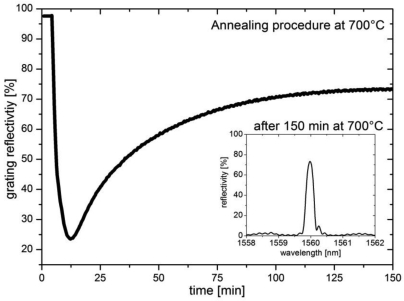
Annealing behavior of the grating reflectivity at 700 °C.

In Figure 1, the annealing behavior of a grating written with an energy density of 700 mJ/cm^2^ and a resulting fluence of approximately 2 kJ/cm^2^ is shown. The grating has a transmission loss of −16.0 dB, which corresponds to a reflectivity of 97.5%. The spectral width was measured to be 0.381 nm (FWHM), and its Bragg wavelength at room temperature was 1,550.670 nm. The grating was then annealed at 700 °C after it was measured at room temperature for the first four minutes.

The picture shows the evolution of the grating reflectivity, which is representative for the UV-induced refractive index modulation during isothermal annealing. The grating reflectivity decreases during the first 10 minutes before a secondary growth in grating reflectivity and so a refractive index modulation is observed.

In contrast to Canning *et al.* the regeneration process occurs at lower temperatures and without applying hydrogen to the fiber. Furthermore the UV laser wavelength and the composition of the fiber differ from our case. In [1,2] a fiber doped with boron and germanium in the core and phosphorous and fluorine in the inner cladding was used, which has a different glass softening point and maybe different stress profiles. Besides the composition, the exposure conditions during grating writing play a major role due to the regeneration process [3]. The mechanism which is responsible for the second increase of refractive index is currently not completely understood and could not be explained with the assumptions in [1,2]. A possible explanation of this effect is crystallization in the doped material caused by UV radiation together with the thermal heating. This could induce a secondary grating reflectivity maximum, which is reached after 150 min of heating. This secondary growth leads to an index change in the fiber core which is more temperature-stable than normal Type I Bragg gratings. The inset graph in Figure 1 shows the spectrum of the grating after 150 min at 700 °C. The regenerated reflectivity was 74 % and its spectral width (FWHM) was about 0.26 nm. After annealing at 700 °C, the gratings can be used at temperatures up to 600 °C, without showing any significant drift or hysteresis. In combination with a flexible interferometric writing technique one can in this way generate multiple Bragg gratings for high-temperature sensor networks.

## 3. Results of Grating Arrays

As an example, an array of four gratings with wavelengths in the telecommunication C band was generated in photosensitive fiber by using a modified Talbot interferometer configuration [5,6]. The wavelengths were chosen from 1,530 nm to 1,545 nm at 5 nm intervals for avoiding spectral overlap in case of possibly different temperatures. A spatial distance of 10 mm between the gratings was chosen, but any other distance is also possible.

The gratings were annealed at 700 °C for around three hours until the maximum regeneration was reached (as in Figure 1). The number of the gratings in an array is not limited and depends only on the measuring equipment (light source, interrogator) and the available fiber length.

**Figure 2.**
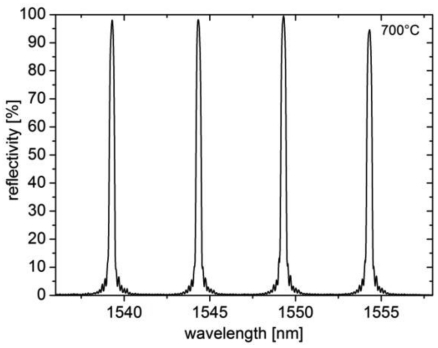
Spectrum of four gratings with maximum regeneration of reflectivity at 700 °C.

**Figure 3.**
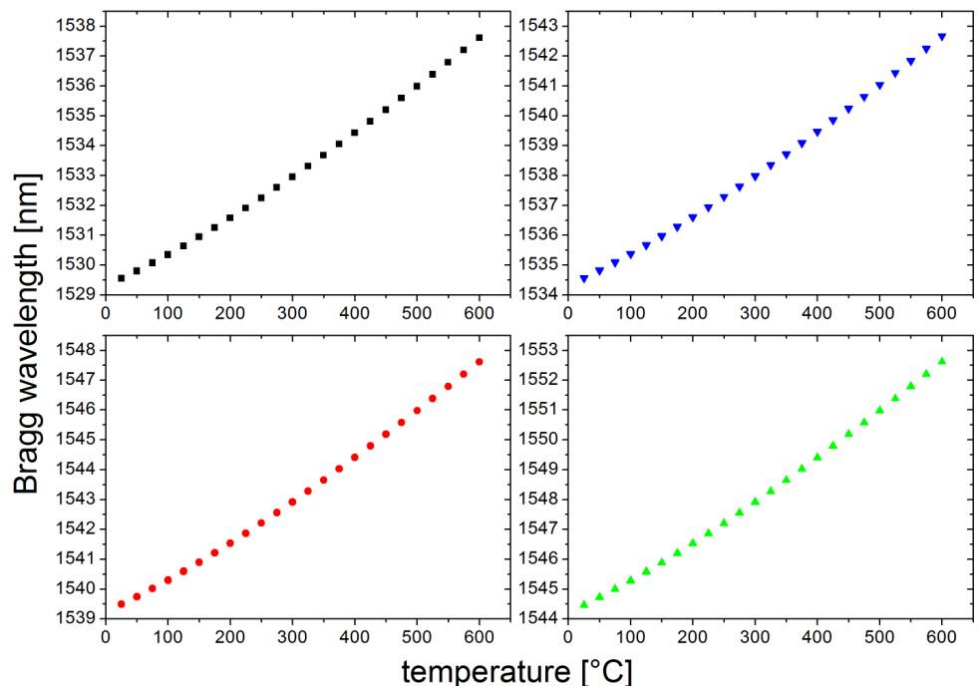
Temperature calibration from 25 °C to 600 °C and back for the four gratings shown in Figure 2.

The grating spectra at 700 °C are shown in Figure 2. All gratings have reflectivities of more than 90% and a spectral width (FWHM) of around 0.3 nm after regeneration. This array was then calibrated by using a temperature calibrator (Fluke 9173) from room temperature up to 600 °C. The results are shown in Figure 3. The four gratings were heated from 25 °C up to 600 °C and cooled again in steps of 25 °C for 20 min. The calibration curves show no significant drift or hysteresis during the heating. The observed deviations in the range of only 5 pm correspond to temperature errors less than 0.5 °C over the whole temperature range.

## 4. Conclusions

We have demonstrated the possibility of using a thermally induced regenerative process in fiber Bragg gratings to increase their stability for temperatures up to 600 °C. For this purpose we have used highly photosensitive fibers without hydrogen loading. Together with an interferometric writing method it is possible to generate arrays of temperature-stable grating arrays for sensor networks. The resulting Bragg gratings combine the attributes of good spectral shape and high reflectivity with high temperature stability showing no drift or hysteresis. Gratings of this type are eminently suitable for temperature sensing networks up to 600 °C.
